# The pediatric oral mycobiome: a comprehensive review of its role in health and disease

**DOI:** 10.3389/fcimb.2026.1711789

**Published:** 2026-01-28

**Authors:** Zhenting Xiang, Yuan Liu

**Affiliations:** Laboratory for Oral Health and Translational Research, Department of Oral Health Sciences, Maurice H. Kornberg School of Dentistry, Temple University, Philadelphia, PA, United States

**Keywords:** children, interspecies interactions, next generation sequencing, oral disease, oral mycobiome

## Abstract

The oral microbiome functions as an intricate and coordinated microbial network, residing throughout the oral cavity in both health and disease. Although most oral microbiome research has focused on bacteria, there is a growing interest in oral fungal communities, known as the oral mycobiome. The oral cavity hosts a complex and diverse mycobiome comprising of an estimated 100 fungal species; however, the roles of these fungi have been largely overlooked and remain insufficiently characterized, particularly in children. This represents a critical gap, as early life is a key window for establishing oral microbial communities that shape lifelong oral and systemic health and offer opportunities for early intervention. Recent technological advances, especially next-generation sequencing, have enabled the identification of new fungal species and deepened our understanding of the diversity, structure, and interactions among fungal, bacterial, and other components within the oral cavity. Yet, research on the pediatric oral mycobiome remains fragmented and limited in scope. Addressing this gap is important since the early-life oral mycobiome may play an underappreciated role in shaping immune development, influencing susceptibility to oral diseases, and potentially contributing to systemic conditions during childhood and beyond. In this review, we examine the oral mycobiome in children, focusing on its formation and dynamics in health and in disease, including dental caries, periodontal disease, endodontic infection, and cleft lip/palate, and exploring its connections to several systemic consequences. By synthesizing current findings on fungal-related biological risk factors, we aim to inform the development of improved diagnostic tools and to guide the advancement of preventive and therapeutic strategies from fungal perspective.

## Introduction

The oral cavity consists of both hard and soft tissues and maintains a moist environment that connects it to the external surroundings (Li et al., 2022). Given the complexity of its anatomical structure, the oral cavity harbors a diverse and intricate ecosystem consisting of various microorganisms, including millions of bacteria, viruses, and fungi (Montelongo-Jauregui and Lopez-Ribot, 2018). The multidirectional interactions and dynamics between this polymicrobial community, known as the oral microbiome, their genomes, metabolites, and the human host ultimately affect health and disease states, especially in children (Jenkinson and Lamont, 2005; Gomez and Nelson, 2017; Sedghi et al., 2021). Since the launch of the Human Microbiome Project, most research efforts and interest have focused on bacterial microbiota, however, an indispensable component of the microbiome is the mycobiome, specifically referring to the fungal community (Limon et al., 2017; Bandara et al., 2019). In pediatric oral health, research has similarly focused largely on bacterial contributors to diseases, while the oral mycobiome has received comparatively limited attention until recent years (Diaz et al., 2017; Diaz and Dongari-Bagtzoglou, 2021).

Similar to the gastrointestinal tract, skin, respiratory tract and other mucosal surfaces where diverse fungi are found (Limon et al., 2017), the oral cavity also contains a community of commensal fungal organisms, with *Candida* as the most commonly detected genera (Ghannoum et al., 2010; Peters et al., 2017; Rajendra Santosh et al., 2021). Many of these fungal species naturally inhabit healthy individuals, with 35% or more of healthy individuals harboring these microbes maintaining a homeostatic balance and a symbiotic relationship with the human host (Lalla et al., 2013). In early life, when microbial and immune systems are still developing, the balance is especially delicate. Disruption of the oral mycobiome can contribute to dysbiosis, which has been linked to various disease states. For example, oropharyngeal candidiasis (oral thrush), often occurs when this ecological equilibrium is disturbed (Rajendra Santosh et al., 2021). Children may be especially susceptible to such disturbance due to age-specific host factors and vulnerabilities. Predisposing factors such as the use of orthodontic appliances or a history of cleft palate can increase the colonization and persistence of several fungal species (Lalla et al., 2013). Additionally, interactions between fungi, bacteria, and other microbial components add further complexity to the developing oral ecosystem, influencing not only the onset and progression of oral diseases, but also children’s response to treatment (Diaz et al., 2014).

**Figure 1:**
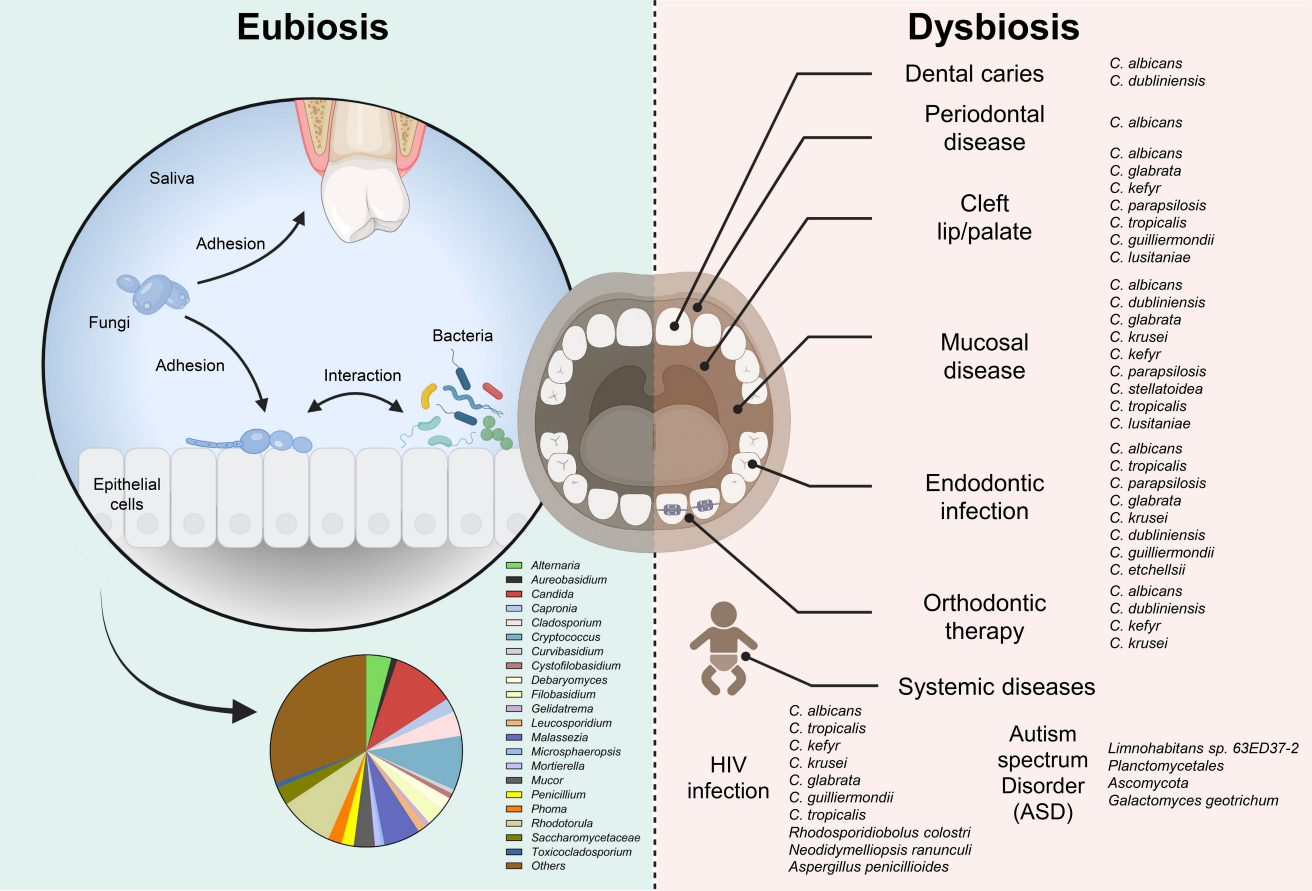
Complex fungal populations, encompassing both eubiosis and dysbiosis, are closely associated with oral health and disease. The figure illustrates representative patterns of fungal community composition observed under healthy (Fechney et al., 2019) versus diseased conditions. For each disease state (on the right side), only those species are listed for which current studies have reported associations between changes in their abundance or presence and the corresponding clinical condition. Created with BioRender.com.

Exploring the functional role of oral fungi and their interkingdom interaction with oral bacteria and the host immune system is essential for understanding disease pathogenesis and identifying new therapeutic opportunities. Despite rapid advances in microbiome research, pediatric populations remain understudied, particularly with respect to fungal dynamics that may shape lifelong oral and systemic health. The oral mycobiome is also largely overlooked in the development of microbiota-based therapies, and current findings have yet to be fully translated into clinical practice. In light of these gaps, this review synthesizes recent progress in basic and translational research on the pediatric oral mycobiome (**Figure 1**). We highlight emerging evidence on the functional contributions of fungi to oral diseases, as well as the ecological effects of bacterial-fungal interactions on host physiology and pathophysiology. Finally, we discuss the potential clinical implications of oral mycobiome research and outline the opportunities for translating fungal-focused insights into diagnostic tools and preventive strategies, with the goal of improving early identification of biological risk factors and advancing precision oral health care for children.

## Commensal mycobiome in children

The microorganisms residing in the oral cavity, and their inevitable inter-relationships, are intricately linked to the balance between health and disease, not only locally but also systemically (Peng et al., 2022). In a healthy individual, the host immune response is able to properly balance inflammation, allowing the removal of potentially harmful microorganisms while preventing inappropriate immune responses against commensal microorganisms that are essential for maintaining health (Sultan et al., 2018). Studies have elucidated a symbiotic relationship between the host and its resident microorganisms. This equilibrium, called “eubiosis” or “microbial homeostasis”, is mutually beneficial; the host offers a stable ecological niche for these commensal microbes, which in turn have long-term consequences for health (Radaic and Kapila, 2021; Tuganbaev et al., 2022). Disruption of this homeostasis of the microbiome (“dysbiosis”), which can occur when commensal microorganisms are lost or diversity is lost, is increasingly recognized as a contributing factor in the pathogenesis of various oral and systemic diseases (Petersen and Round, 2014; Radaic and Kapila, 2021; Baker et al., 2023). Therefore, understanding the composition and function of a healthy oral microbiome is important to grasp its significance in human health.

In comparison to bacteriome research, relatively little effort has been dedicated to characterizing the fungal community in health. Despite the ever-rising incidence of fungal infections, particularly in association with underlying immunodeficiencies, the diversity and functionality of resident fungi have been largely overlooked (Bandara et al., 2019). The reasons are manifold; it is difficult to sequence low human-associated fungal biomass and fungal cultivation issues prevent reference sequence generation (Underhill and Iliev, 2014; Suhr and Hallen-Adams, 2015). Additionally, metagenomic sequencing has not been adopted for fungi, due to the lack of a good, culture-independent method of separating fungi from the predominant bacteria (Suhr and Hallen-Adams, 2015). Other reasons include lack of quality-controlled reference databases for fungi, difficulties in nomenclature for fungi and incorrectly annotated fungal databases (Ward et al., 2017; Bandara et al., 2019). These challenges further point to the importance of novel approaches to gain robust and accurate mycobiome characterization.

Over the past decade, accumulating studies have provided insight into the landscape of gut mycobiome composition in humans (Nash et al., 2017; Zhang et al., 2022), including keystone fungal species that are fundamental to human health or linked to disease onset and progression. Fungal species that assemble in the gut may represent an important aspect of human biology even before birth (Willis et al., 2019). Studies have found that infants harbor a gut mycobiome predominated by the orders *Saccharomycetales* and *Malasseziales* between 1 to 4 months of age, followed by a gradually diminish of *Malasseziales* to an undetectable level (Fujimura et al., 2016). With the dietary shift to solid food, *Saccharomyces cerevisiae* becomes the most abundant species (Schei et al., 2017), and then, the gut mycobiome undergoes further maturation towards a stable adult-like fungal community that features with substantially increased diversity. By 1 year of age, the gut fungal community of a child is largely established, and this may have beneficial effects against allergies and gastrointestinal disease later in life (Rodriguez et al., 2024). Known mycobiome-modifying factors in early life include maternal diet, delivery mode, feeding mode, mother’s microbiota, gestational age at birth, antibiotic exposure, and bacterial microbiome (Boutin et al., 2021; Auchtung et al., 2022; Turunen et al., 2023; Mercer et al., 2024; Rodriguez et al., 2024). As the infant grows, mycobiota diversity is further impacted by environment, cultures, and geographic locations; however, it is unknown if these differences persist in later life, which requires large long-term longitudinal studies. With the advancement of sequencing technologies, there is an increased interest in mycobiome studies; however, limited research on the mycobiome and child health outcomes has resulted in a huge knowledge gap in this field.

The oral cavity, as an important route of entry of the body, is colonized by a wide diversity of fungi (Diaz and Dongari-Bagtzoglou, 2021). These studies usually report hundreds of fungal taxa, suggesting a highly diverse mycobiome could play an essential role in the oral microbiome by interacting with other components in the community and adjacent teeth and mucosal tissues. One of the pioneering studies conducted by Ghannoum et al., described the basal oral mycobiome in healthy individuals (Ghannoum et al., 2010). In total, 101 different fungal species in the oral samples were detected. Among them, *Candida* species emerged as the most prevalent, found in 75% of the participants, followed by *Cladosporium* (65%), *Aureobasidium* (50%), *Saccharomycetales* (50%), *Aspergillus* (35%), *Fusarium* (30%), and *Cryptococcus* (20%) (Ghannoum et al., 2010). In healthy individuals, *Candida* or *Malassezia* are the most common fungal genera, with *Candida* metabolizing carbohydrates and *Malassezia* utilizing lipids (Baker et al., 2023).

Although there is a lack of research on the oral mycobiome specific to children, it is still important to explore this region of the mycobiome when examining any individual’s fungal composition. From an early stage of life, the human oral cavity encounters a wide variety of microorganisms. These early microbial communities have therefore a major impact on organizing and shaping the adult microbiome, and may present a source of protective and pathogenic microorganisms in early stages of life. Colonization of oral mucosal surfaces begins at birth with the introduction of bacteria and fungi through multiple paths, including maternal transmission, parental exposures, diet and horizontal transmission (Xiao et al., 2020).

Previous exploration of the oral mycobiome of infants has been restricted to the use of *Candida*-specific culturing, demonstrating the presence of numerous *Candida* species, such as *C. albicans*, *C. parapsilosis*, *C. krusei*, *C. guilliermondii*, *C. dubliniensis*, and *C. tropicalis* (Kleinegger et al., 1996). Similarly, a studied using internal transcribed spacer 2 amplicon sequencing found *C. parapsilosis*, *C. tropicalis*, and *C. orthopsilosis*, as well as *Saccharomyces cerevisiae* and *Cladosporium velox*, to be the most relatively abundant and prevalent fungal species in the infant oral mycobiome during the first month of life (Ward et al., 2018). The abundance of *Candida* is age-dependent; its relative abundance is lower at birth and rises with age in the first year of life (Bandara et al., 2019). As the infant ages and transitions to a more adult-like diet, the mycobial community reaches a mature and diverse state.

## Oral mycobiome and childhood caries

Dental plaque is a biofilm that consists of bacteria, fungi and other microorganisms organized in a matrix of extracellular products, and the highly structured nature of dental plaque allows microorganisms to interact with one another and provides protection against host defenses (Demuyser et al., 2014). ITS-based studies suggested that certain fungi, such as *Debaromyces*, *Rhodotorula*, or *Malassezia*, which are prevalent in caries-free children, might play a role in antagonizing cariogenic bacteria (Baraniya et al., 2020; O’Connell et al., 2020). Researchers also found that caries influences the abundance of specific fungi, where children without caries had a significantly higher abundance of 17 species compared to children with caries (Fechney et al., 2019). Moreover, the interplay between oral bacteria and fungi in healthy children is crucial for the stability of the oral cavity’s microbiome (Radaic and Kapila, 2021). Typing analyses of salivary samples revealed distinct fungal profiles across children with different health conditions and identified health-related cluster. Notably, *Malasseziales* and *Neocosmospora* were negatively correlated to caries indices. Spearman correlation analysis further showed a significant inverse association between *Fusobacterium* and *Neocosmospora*, suggesting opposing relationships with maintaining caries-free status and caries development (Tu et al., 2022). However, the study by Cheung et al. reported limited bacterial-fungal interactions in the healthy oral microbiota, with the most significant association observed between *Pseudomonas* sp. and *Rhodotorula dairenensis* (Cheung et al., 2022). These interactions are critical as they can enhance the virulence of these microorganisms and potentially reduce their susceptibility to host defense mechanisms.

*Streptococcus mutans* is the most common bacterium associated with the pathogenesis of dental caries, and there are several characteristics of the species that aids in its pathogenicity; *S. mutans* is able to thrive in acidic environments; it is able to create extracellular polymeric substance (EPS), a matrix that allows dental plaque to form, and its ability to metabolize dietary carbohydrates into acid (Lemos et al., 2019). Recent studies have demonstrated that *S. mutans* may not be the only pathogen that increases a child’s risk of dental caries, as several studies have shown that an increased prevalence or abundance of *Candida* in the oral cavity of children may be associated with early childhood caries (ECC) (Lozano Moraga et al., 2017; Alkhars et al., 2022; Cvanova et al., 2022). One study has shown that the presence of *C. albicans* in a multispecies biofilm induces the expression of particular glycosyltransferase genes, which are involved in EPS formation (Falsetta et al., 2014). This study also noted that in these mixed biofilms, genes involved in acid tolerance are also upregulated (Falsetta et al., 2014). This furthers the notion that interactions between *C. albicans* and *S. mutans* may be involved in the initiation and progression of ECC. However, one author has argued that *C. albicans* can actually prevent the progression of dental caries by increasing the pH of the oral environment (Willems et al., 2016).

The role of the oral mycobiome in the development of dental caries in children has been a focus of recent research studies. These studies have investigated the relationship between the prevalence of oral fungi and dental caries in children. Several of these studies have reported a positive correlation between the presence of *C. albicans* and caries in children. Alkhars et al. followed socioeconomically disadvantaged children from birth to age 2, showing that infants who were bottle fed within the first 4 months after birth had a higher prevalence of *C. albicans* compared to infants who were breast fed or both breast and bottle fed (Alkhars et al., 2022). The study also found that an increased maternal carriage of *C. albicans* and *S. mutans* is positively associated with infants’ carriage of *C. albicans*. In addition, in infants with early colonization of the oral cavity by *C. albicans* there was a 3.5 times higher emergence of *S. mutans* (Alkhars et al., 2022).

*C. albicans* was not the only species noted within recent studies, as the prevalence of *C. dubliniensis* was highlighted as high in children with severe ECC (S-ECC) (Lozano Moraga et al., 2017; O’Connell et al., 2020; Cvanova et al., 2022; Tu et al., 2022). Lozano et al. found that *C. albicans* was the most prevalent species of *Candida* in children with ECC, while *C. dubliniensis* was only found in the groups of children with the most severe caries (Lozano Moraga et al., 2017). In fact, *C. dubliniensis* is mainly found in the presence of caries and at relatively low levels within caries-free children, which poses a connection of *C. dubliniensis* as an ECC indicator species compared to *C. albicans* (de Jesus et al., 2020; O’Connell et al., 2020). However, the focus of fungal involvement in ECC has been reliant on *C. albicans*, and it begs the question if the close relationship with *C. dubliniensis* may have masked its presence as *C. albicans*. The overlap of both species overlooks the role of *C. dubliniensis*’ role in ECC. Thus, it is necessary to provide detailed sequencing to distinguish between *C. albicans* and *C. dubliniensis* (O’Connell et al., 2020).

While the majority of studies identified *Candida* as the primary fungal genus in childhood caries, Yin et al. discovered *Cryptococcus* as the most abundant genus (Yin et al., 2023). Along with *C. albicans*, other S-ECC-related fungi discovered in abundance were *Cryptococcus neoformans*, *Candida sake* and *Nigrospora oryzae* (Cui et al., 2021). Other species, such as *C. glabrata, C. tropicalis*, *C. guilliermondii, C. krusei*, and an unclassified *Microdochium* sp. were also correlated with caries in different studies (Man et al., 2025).

The mechanisms contributing to the impact of *Candida* on development of ECC are not fully understood, however some studies have begun to analyze the complex interkingdom interactions that play a role in the development of ECC, such as that done by Tu et al (Tu et al., 2022), which identified a significant correlation of cross-kingdom taxonomic pairs. However, this study has been limited to the genus level due to the depth of sequencing available (Tu et al., 2022), showing us that more in depth and advanced analyses will need to be done in the future in order to completely understand the interactions between the bacteriome and mycobiome as it relates to caries risk in children. Interkingdom correlation analysis reveals that *C. dubliniensis* is strongly correlated with both *Neisseria bacilliformis* and *Prevotella veroralis* in ECC (Khan et al., 2024), and the adhesins in the *Neisseria* genus may help facilitate the co-existence of *Neisseria* and *Candida* species (de Jesus et al., 2020).

## Oral mycobiome and endodontic infection in children

For decades, studies have shown that the microbiota associated with endodontic infections comprise a complex mixture of bacterial species. The species most frequently found in the pulp chamber of primary teeth are *Porphyromonas gingivalis*, *Prevotella intermedia*, *Porphyromonas nigrescens*, and *Fusobacterium alocis*. Frequently found microbial species in root canals of primary teeth were *P. gingivalis*, *P. intermedia*, *Actinomycosis naeslundii*, *P. nigrescens*, and *Treponema forsythia* (Gomes et al., 2013). *Enterococcus faecalis*, most commonly recovered from teeth with failed endodontic therapy, was recently also found in primarily infected teeth (Gomes et al., 2006; Cogulu et al., 2007). Recently, A handful of studies showing that the occurrence of fungi in endodontic infections have sparked a renewed interest in the role of these mycobiome, especially *Candida* species, in resultant of endodontic sequelae (Siqueira and Sen, 2004; Alberti et al., 2021). *Candida* species have been found in infected permanent tooth root canals ranging from 0.5%–55% (Mergoni et al., 2018), with *C. albicans* as the most prevalent species. *C. tropicalis*, *C. parapsilosis*, *C. glabrata*, *C. krusei*, *C. dubliniensis*, *Candida guilliermondii*, and *Candida etchellsii* have also been detected in infected root canals (Mergoni et al., 2018). These yeasts can survive within the harsh and barren ecosystem of the root canals. Moreover, they can produce hydrolytic enzymes, undergo morphologic transition, form biofilm, and evade and modulate the host defense. Furthermore, it can synergistically interact with other microorganisms and enhance pathogenicity. A study showed that co-cultured *C. albicans* and *Enterococcus faecalis*, which is highly prevalent in failed endodontic treated root canal, demonstrated mutually enhanced growth and improved survival of detrimental stresses including alkalinity starvation, mechanical shear force, and antimicrobials such as sodium hypochlorite and chlorhexidine (Gao et al., 2016). In addition, co-culture with these taxa induced increased IL-6 and TNF-α responses and caused significantly more extended periapical lesions in an animal model (Du et al., 2021). Currently, the role of other fungal species in endodontic infections remains to be clarified and represents an important research line in the field.

It is noteworthy that vast majority of these studies on fungal involvement in endodontic pathology have been conducted in permanent teeth with primary or secondary endodontic infections in adult patients, resulting in a significant gap in evidence regarding the fungal biome of primary teeth and immature permanent teeth with pulp infection in children. Only a limited number of pediatric-focused investigations exist. For example, Ledezma-Rasillo et al. (2010) evaluated cultivable microorganisms from 21 primary teeth with necrotic pulps and detected *C. albicans* in only 3 cases (6.25%). There is virtually limited information on how fungi interact with bacteria in the developing dentition, host age and pulp immunology influence endodontic infection in pediatric populations. Future research is needed to incorporate sequencing-based technologies, *in vitro* organ-on-chip models, and *in vivo* animal studies to comprehensively characterize the pediatric endodontic mycobiome, polymicrobial interactions, and age-specific immune response in pulp infection. Mechanistic *in vitro* and *in vivo* models tailored to primary and immature teeth would help clarify functional roles of fungi in pulp inflammation, necrosis, and periapical pathology.

## Oral mycobiome and periodontal disease in children

In the pediatric population, gingivitis is very common and usually associated with plaque accumulation. It is reversible with proper oral hygiene (Pawlaczyk-Kamieńska et al., 2018). In contrast, periodontitis, although less frequent, assumes clinical significance, especially in children with systemic, genetic, or immunological conditions, where it is often presented as an aggressive and destructive condition (Kinane et al., 2001). Periodontal diseases result from a complex interplay between microbial dysbiotic biofilms and the host immune inflammatory response, influenced by genetic and environmental factors (Radaic and Kapila, 2021). In gingivitis and periodontitis, the microbial composition shifts toward dysbiosis, characterized by an increase in pathogenic bacteria belonging to the red and orange complexes, such as *P. gingivalis*, *Tannerella forsythia*, *Treponema denticola*, and *Fusobacterium nucleatum* (Lamont et al., 2018). *Aggregatibacter actinomycetemcomitans* has been strongly associated with periodontitis in pediatric populations due to its virulence factors, such as leukotoxins that induce neutrophil apoptosis and promote inflammation (Fine et al., 2019). Beyond the role of bacteria, emerging evidence has highlighted the potential role that certain fungi, may attribute to the pathogenesis of periodontal disease through synergistic interactions with bacterial pathogens which may exacerbate bacterial tissue invasion and inflammation (Suresh Unniachan et al., 2020). Several studies found that *C. albicans* is the most prevalent fungus in the periodontal pocket of periodontitis patients (Urzúa et al., 2008; Al Mubarak et al., 2013; Canabarro et al., 2013; De-La-Torre et al., 2018). Co-infection with *C. albicans* and *Porphyromonas gingivalis* has also been confirmed to be significantly associated with deep periodontal pockets and bleeding, contributing to active periodontitis (Oka et al., 2022). *In vitro* studies further demonstrate that *C. albicans* and *P. gingivalis* dual-species biofilm exacerbates periodontal disease, with increasing epithelial cells invasion by *P. gingivalis*. *C. albicans* may serve as a scaffold to allow *P. gingivalis* sufficient time for invasion (Tamai et al., 2011).

Children with primary immunodeficiency diseases often have oral manifestations, including aggressive forms of periodontal disease. Among these conditions, severe congenital neutropenia and leukocyte adhesion deficiency are associated with pronounced periodontal breakdown, and the predominant fungal species detected in periodontal pockets in these patients is *C. albicans* (Halai et al., 2020). Similarly, *C. albicans* has been detected in periodontitis associated with Fanconi anemia in children (Nowzari et al., 2001). Despite these observations, the underexplored role of fungi, particularly the oral mycobiome, in the pathogenesis of periodontal disease in systemically compromised children remains poorly characterized. Existing studies are limited by small sample sizes, a reliance on culture-based methods that underestimate fungal diversity, and cross-sectional designs that preclude determination of causality. Moreover, many investigations focus exclusively on *C. albicans*, failing to capture the complexity of the broader fungal community and its potential interactions with bacterial pathogens, host immune dysfunction, and environmental factors.

## Oral mycobiome and orthodontic therapy in children

Placement of biomaterials in the oral cavity, such as orthodontic appliances, can perturb the oral microbiome by creating plaque-retentive niches. Appliance insertion increases surface area and stagnation zones that favor biofilm accumulation and shift toward cariogenic and periodontopathogenic taxa. These microbial changes often occur alongside appliance-associated alternations in the local environment, including chemical and biological conditions, salivary parameters, diet, and related lifestyle behaviors. Consequently, orthodontic appliances have been associated with elevated caries risk and exacerbation of pre-existing periodontal inflammation (Thornberg et al., 2009; Lucchese and Bondemark, 2021; Campobasso et al., 2022). Studies in pediatric populations suggest a positive correlation between orthodontic therapy and the presence of multiple *Candida* spp., a shift linked to increased risk of periodontal disease (Arendorf and Addy, 1985; Rodríguez-Rentería et al., 2021).

Children wearing fixed orthodontic appliances typically show greater increases in oral colonization by both bacterial (e.g., *S. mutans*, *Lactobacillus* spp.) and fungal taxa, particularly *Candida* species (Khanpayeh et al., 2014; Yang et al., 2022; Siripaisanprasert et al., 2025). Data indicate a stronger propensity for colonization with non-*albicans Candida* in these patients compared to those with removable appliances, who show a predominance of *C. albicans* (Campobasso et al., 2022). Removable orthodontic appliances also raise *Candida* colonies, especially *C. albicans* species during the first month of therapy, but followed by a decrease after a few months (Kundu et al., 2016; Khanpayeh et al., 2014). Similarly, authors reported a decrease in the normal level of the microbiota and an increase in the frequency of *C. albicans* together with *S. aureus* and *S. mutans* (Zharmagambetova et al., 2017).

Multiple investigations confirm that longer appliance wear, especially over 3 to 6 months, increases oral *Candida* colonization, with emerging presence of new strains such as *C. dubliniensis, C. kefyr, and C. krusei* during treatment (Brzezińska-Zając et al., 2023; Siripaisanprasert et al., 2025). One prospective study found that the proportion of children free from *Candida* decreased from 70% at treatment initiation to 41% after six months of removable appliance use (Brzezińska-Zając et al., 2023). Similarly, salivary *Candida* colony counts rise in both fixed and removable appliance users (Khanpayeh et al., 2014). However, evidence for appliance-associated shifts in the broader oral mycobiome remains limited, with most available data focused on *Candida* rather than non-*Candida* fungi.

Despite the increased colonization, overt clinical manifestations of oral candidiasis remain rare in healthy children, suggesting a degree of host resistance. However, given the ecological shifts and potential risks, clinicians are advised to emphasize personalized oral hygiene protocols and monitor periodontal health throughout orthodontic treatment. The impact appears most pronounced in the initial stages of treatment, supporting early preventive strategies (Mulimani and Popowics, 2022; Peterson et al., 2024).

## Oral mycobiome and cleft lip/palate in children

Orofacial cleft disorders, including cleft lip and/or palate (CL/P), are among the most common congenital disorders worldwide. Various dental conditions, such as enamel hypoplasia, asymmetrical development of the dentition, and microdontia, are also associated with CL/P (Gershater et al., 2023). CL/P has been shown to influence the composition and ecological balance of the oral microbiome. Rawashdeh et al. investigated the prevalence of *C. albicans* and other *Candida* species in children and adolescents with clefts across three age groups, compared with healthy controls (Rawashdeh et al., 2011a). Their findings demonstrated a significantly higher rate of oral *Candida* colonization in CL/P patients, with an overall asymptomatic carriage rate of 63.3%, most commonly *C. albicans*, followed by *C. glabrata*, while *C. kefyr* was the least frequent. In contrast, 18.3% of control participants harbored *Candida*, and only *C. albicans* and *C. kefyr* were detected. It is worth noting that although *C. albicans* is by far the most commonly investigated fungal species in studies of CL/P patients, reported detection rates vary widely-from below 10% to nearly 70% (Mattos et al., 2009; Rawashdeh et al., 2011b; Machorowska-Pieniążek et al., 2017; Yilmaz et al., 2020; Boriollo et al., 2022). Interestingly, Boriollo et al. observed a higher prevalence of non-*albicans Candida* species, particularly *C. krusei* and *C. tropicalis*, in individuals with CL/P compared with healthy controls (Boriollo et al., 2022).

Children with palatal clefts can have reduced middle ear ventilation due to incorrect insertion of the palate muscles, which often prevents the Eustachian tube from fully opening (Flynn et al., 2009). This can lead to effusions, causing dampened sound conduction with hearing impairment and potential infections. In a retrospective study, Kakoschke et al. analyzed 142 swab samples collected from 112 children with clefts and otitis media with effusions, and detected *Candida* in the exudate of 23 swabs (16.2%) from 19 patients (16.9%) (Kakoschke et al., 2025). *C. albicans* was detected in 53.8% of cases, *C. parapsilosis* in 34.6% of cases and *C. tropicalis*, *C. guilliermondii* and *C. lusitaniae* in the remaining 11.7% of cases. Overall, these findings highlight substantial variability in the oral *Candida* of CL/P patients and illustrate the need to broaden research beyond *Candida* to better understand the microbial ecology associated with orofacial clefts.

## Oral mycobiome and mucosal disease in children

Although numerous mycobiome surveys have been conducted, *Candida* remains the only fungal genus clearly implicated in the etiology of the most common oral mucosal infection. Oral candidiasis (OC), or “thrush”, affects approximately 5% of newborns, increasing to 14% by four weeks of age (Boscarino, 2023). *C. albicans* is the predominant causative species, responsible for up to 95% of OC cases (Vila et al., 2020). Other *Candida* species reported in various study populations include *C. dubliniensis*, *C. glabrata*, *C. krusei*, *C. kefyr*, *C. parapsilosis*, *C. stellatoidea*, and *C. tropicalis* (Millsop and Fazel, 2016). OC is also highly prevalent in children undergoing cancer treatment, particularly during episodes of severe neutropenia. In a study by Alberth et al., 23 of 30 pediatric oncology patients (76.7%) who developed moderate-to-severe neutropenia, had oral fungal colonization or clinically obvious fungal infection (Alberth et al., 2006). While *C. albicans* was the initial pathogen detected, prolonged neutropenic stage was associated with the emergence of non-*albicans Candida* species, including *C. kefyr*, *C. lusitaniae*, *C. sake*, and *C. tropicalis* (Alberth et al., 2006). Similarly, González-Gravina et al. reported that *C. albicans* was the most frequently isolated species from children with cancer, followed by *C. parapsilosis*, *C. tropicalis*, *C. krusei*, *C. glabrata* and *C. lusitaniae* (González Gravina et al., 2007). In some cases, two different species were identified in the same patients, i.e., *C. albicans* and *C. tropicalis* (2 patients); *C. albicans* with *C. krusei* (1 patient), and *C. tropicalis* and *C. parapsilosis* (1 patient) (González Gravina et al., 2007). These studies highlight not only the dominant role of *C. albicans* in pediatric oral mucosa infections but also the increasing relevance of non-*albicans Candida* species in immuno-compromised children.

## Oral mycobiome and systemic diseases in children

In 1891, W. D. Miller, the first oral microbiologist, proposed the theory of oral focal infection, suggesting that microbial infections in the oral cavity could affect distant organs and contribute to systemic diseases (Miller, 1891; Peng et al., 2022). Although this early concept did not receive sufficient attention at that time, advancements in microbiome research have profoundly reshaped our understanding of the oral cavity’s role in whole-body health. Evidence linking the oral microbiota to systemic diseases and overall health continues to accumulate. Contemporary research demonstrates that the oral microbiome is not an isolated ecosystem but a dynamic microbial community capable of influencing a wide spectrum of systemic conditions, including cardiovascular disease, diabetes, adverse pregnancy outcomes, autoimmune disorders, and neurodegenerative diseases (Baker et al., 2023). Mechanistic studies increasingly show that oral pathogens and their metabolites can disseminate through the bloodstream, modulate host immunity, and induce chronic inflammation, thereby contributing to disease onset or exacerbation. Moreover, systemic disease states often induce gradual, reproducible shifts in the oral microbial community, reflecting alterations in host physiology. These consistent microbial signatures highlight the oral microbiome as a sensitive, real-time indicator of human health.

Asthma is the most common chronic disease in children, and asthma attacks (exacerbations) pose significant risks to their health (Pijnenburg and Fleming, 2020). The underlying mechanisms driving asthma in children remain incompletely understood, stemming from a complex interplay of various factors, such as genetic predisposition, environmental exposures, and microbiota. In recent years, there has been growing interest in the oral microbiome as a potential contributor to asthma pathogenesis and management. While research has centered on the lower airway and gut microbiota, emerging evidence highlights the oral cavity as a dynamic microbial reservoir that both reflects and influences the respiratory health (Liu et al., 2025). The oral cavity serves as a significant source of microbes that are regularly aspirated into the lungs, where they can modify the pulmonary microenvironment (Segal et al., 2016). This microbial transfer plays a crucial role in shaping local immune responses, potentially influencing the progression and severity of asthma (Wu et al., 2021). Oral dysbiosis has been linked to gut and systemic inflammation, both of which are highly relevant to asthma pathogenesis. For example, Yan et al. reported *Prevotella bivia*, *P. disiens*, *P. oris* and *Bacteroides fragilis* were enriched orally and intestinally in asthmatics, while *Streptococcus thermophilus* decreased. *P. bivia*, *P. disiens* and *P. oris* in asthmatic gut likely originated orally. *P. bivia* increased pro-inflammatory and decreased anti-inflammatory lipid metabolites in human bronchial epithelial cells (Yan et al., 2024). However, relatively few studies have explored the role of the oral fungal community in asthma pathogenesis. Understanding how oral fungal dysbiosis interacts with host immunity and bacterial communities may provide new insights into asthma development and identify novel biomarkers or therapeutic targets for pediatric asthma.

Accumulating evidence has highlighted oral health issues such as periodontitis in adults with diabetes mellitus (DM), which are highly associated with oral bacterial dysbiosis. DM induces changes in connective tissue metabolism, leading to a decreased ability to resolve inflammation and undergo remodeling, which in turn, exacerbates periodontal damage (Xiao et al., 2023). However, in-depth characterization of the oral bacterial community and structural alterations in the oral microbiota at different phases of type 1 diabetes (T1D) remain scarce. T1D is a pediatric-onset disease that results in chronic insulin deficiency and consequent hyperglycemia (Yuan et al., 2022). The prevalence of periodontitis in children with T1D has been reported to be 5 times higher than in non-diabetic controls (Yuan et al., 2022). Similar to findings in patients with type 2 diabetes, the oral microbiota of individuals with T1D also differs markedly from healthy controls, showing increased abundances of genera that include numerous opportunistic pathogens, such as *Streptococcus*, *Actinomyces, Rothia, and Brevundimonas* (Moskovitz et al., 2021). Recently, both animal and human studies suggest that gut fungi influence metabolic health and may contribute to diabetes. In patients with diabetes, the gut mycobiome has emerged as a key contributor to metabolic variation, explaining more of the variance in glycemic traits than bacterial communities or plasma metabolites (Al Bataineh et al., 2023). Studies have reported increased fecal fungal diversity and a marked rise in *Candida* colonization in individuals with T1D (Kowalewska et al., 2016). Similar trends are observed in type 2 diabetes (T2D), where both the richness and evenness of fungal species are elevated compared to healthy controls (Jayasudha et al., 2020). Additionally, opportunistic fungal pathogens such as *Aspergillus* and *Candida* are more abundant in patients with T2D, while the phylum *Mucoromycota* appears to be depleted. These findings underscore the significant and underappreciated role of gut fungi in diabetes pathophysiology (Jayasudha et al., 2020; Bhute et al., 2017). While growing evidence links gut mycobiome alterations to metabolic dysfunction, the oral mycobiome remains largely overlooked, despite the fact that the oral cavity is a key interface between host immunity, diet, and microbial exposure. In children with T1D, who often experience elevated salivary glucose levels and altered immune responses, these changes could create conditions that favor shifts in oral fungal composition, including the overgrowth of opportunistic species such as *Candida*. Understanding how these oral fungal alterations interact with bacterial communities, local inflammation, and systemic metabolic control may provide important insights into disease mechanisms and potential early biomarkers or intervention targets.

Several studies have revealed that the oral microbiome of children living with HIV is distinct from that of children who have never been exposed to the virus. Domaneschi et al. reported a 62% prevalence of oral *Candida* colonization in HIV-infected children (54–72%, 95% CI) (Domaneschi et al., 2011). Among the 86 yeast isolates, *C. albicans* was the most common species (69/86), followed by *C. tropicalis* (5/86), *C. kefyr* (4/86), *C. krusei* (3/86), *C. glabrata* (2/86), and *C. guilliermondii* (1/86) (two samples were not identified due to slower growth) (Domaneschi et al., 2011). Similarly, Cerqueira et al. observed a significantly higher prevalence of *Candida* in HIV-infected children compared with their seronegative siblings, noting that 80% of HIV-infected children (most of whom were not receiving highly active antiretroviral therapy) had positive cultures versus 60% of seronegative controls (Cerqueira et al., 2010). In both groups, *C. albicans* remained the most frequently isolated species, though it was more prevalent in HIV-infected children, whereas *C. parapilosis* was isolated more often from uninfected siblings (Cerqueira et al., 2010). Consistent with these findings, O’Connell et al. also reported *C. albicans* was the most abundant species identified in HIV-infected children, followed by *C. tropicalis*, *Rhodosporidiobolus colostri*, *Neodidymelliopsis ranunculi*, and *Aspergillus penicillioides* (O’Connell et al., 2023). In HIV-infected patients, the transition of *C. albicans* from a commensal member of the oral microbiota to an opportunistic pathogen is driven not only by Th17 CD4+ cell depletion but also by additional mechanisms, including specific binding and interactions between *Candida* and the HIV virus (Cassone and Cauda, 2012). Oral candida infection may spread beyond the mouth to cause esophageal or gastric candidiasis. There is evidence in HIV-infected patients that mycobiome profiles in the respiratory and gastrointestinal tracts are similar to that in the mouth (Cui et al., 2015).

Just as the immunological disruptions in HIV shape oral microbial communities, emerging studies suggest that neurodevelopmental conditions may also be reflected in, and potentially influenced by, oral microbial community. A well-established body of work has already linked the gut microbiota to neuropsychiatric disorders (NPD), including autism spectrum disorder (ASD), through the gut-brain axis. In parallel, an analogue oral microbiome-brain axis has gained increasing attention, proposing that microbes in the mouth may influence neurological function and behavioral outcomes via immune, metabolic, and neuroinflammatory pathways (Bowland and Weyrich, 2022). Several studies examining the oral microbiome profiles in children with ASD consistently report characteristic changes compared with typically developing peers (Hicks et al., 2018; Qiao et al., 2018; Kong et al., 2019; Evenepoel et al., 2024; Tang et al., 2025). For instance, Hicks et al. Identified an increased abundance of *Limnohabitans* sp. 63ED37–2 and *Planctomycetales* in ASD children (Hicks et al., 2018). While these findings highlight important alterations in bacterial components of oral microbiome, the fungal community remains understudied. A recent study conducted by Retuerto et al. compared the gut bacteriome and mycobiome of children with ASD to those of their non-ASD siblings. They found an increase in the fungal *Ascomycota* phylum in ASD patients (Kong et al., 2019). The level of *C. albicans* was also significantly elevated in ASD patients vs. non-ASD sibling controls (27.1% vs. 13.2%) (Kong et al., 2019). In contrast, the abundance of *Galactomyces geotrichum* (17.4% vs. 29.2%) in ASD subjects vs. non-ASD subjects was decreased with a similar decrease in abundance for *Pichia fermentans* (4.4% vs. 6.0%) in ASD vs. non-ASD subjects, respectively (Kong et al., 2019). Both fungal species are primary fermenters and are considered beneficial commensal fungi. Given the parallels between oral and gut microbial disturbances in systemic and neurodevelopmental conditions, future research is essential to elucidate the oral mycobiome’s role in ASD. Such work may reveal novel fungal signatures that not only deepen our understanding of ASD pathophysiology but also offer promising biomarkers for early diagnosis, risk evaluation, and monitoring of disease progression.

## Conclusions and perspectives

The rapid development of high-throughput sequencing and bioinformatics technology has enabled comprehensive investigation of the composition and function of human microbiome. As an important component of this ecosystem, the oral microbiome is both highly structured and influenced by host- and environment-specific factors. However, the predominant focus of microbiome studies has been on the bacterial component, leaving the roles of other microbes, particularly fungi, poorly defined. This gap is especially relevant in early life, when oral health supports nutrition, craniofacial development, immune maturation, and long-term microbial homeostasis. A deep understanding of the pediatric oral mycobiome is therefore crucial, yet current knowledge remains limited. Existing studies often rely on small sample sizes, cross-sectional designs, and methods that insufficiently resolve fungal taxonomy or function. These constraints hinder the identification of true commensal species, the characterization of interactions within the fungal community, and the elucidation of how fungi interface with the host and co-resident bacteria.

Insights from gut mycobiome research indicate that microbial disturbances are rarely the result of a single pathogenic species; rather, they emerge from synergistic interactions among multiple fungal taxa and broader ecological dysbiosis that disrupt homeostasis. Compared with the gut, the study of the oral mycobiome is still in its infancy. Although *Candida* species have justifiably received substantial attention due to their pathogenicity, the pediatric oral mycobiome is taxonomically rich, encompassing genera such as *Malassezia*, *Cladosporium*, *Saccharomyces*, and others, many of which remain understudied. Advancing our understanding of the oral mycobiome carries important clinical implications, particularly for pediatric populations in whom early-life microbial shifts may have long-term health consequences. Furthermore, while microbial dysbiosis is often associated with various oral and systemic disease states, establishing causality remains a major challenge; most studies are observational, and mechanistic pathways remain speculative. Current mechanistic studies using *in vitro* and *in vivo* animal models have elucidated potential mechanisms involved in disease initiation and progression (**Table 1**).

**Table 1:** Potential mechanistic studies exploring roles of fungal species in oral and systemic diseases.

Disease		Fungal species	Methods	Potential mechanism
Dental caries		*C. albicans*	*In vitro* biofilm studies; *In vivo* animal model	*C. albicans* induces *S. mutans* glycosyltransferase genes (EPS production) and upregulates acid tolerance pathways, contributing to potentially more virulent biofilm.
		*C. dubliniensis*	ITS; Interkingdom correlation analysis	Adhesins in *Neisseria* may facilitate co-existence with *Candida.*
Endodontic infection		*C. albicans*	*In vitro* biofilm studies	*C. albicans–E. faecalis* cross-kingdom interaction increases biomass and stress resilience, reducing susceptibility to endodontic disinfection
Periodontitis		*C. albicans*	*In vitro* studies	*C. albicans* may act as a scaffold that facilitates *P. gingivalis* epithelial invasion.
Systemic diseases	Diabetes mellitus	*Aspergillus; Candida; Mucoromycota*	limited	Increased salivary glucose coupled with impaired/altered host immunity may create a permissive niche that shifts fungal community structure toward opportunistic species expansion.
	HIV	*C. albicans*	*In vitro* studies	In addition to Th17 CD4^+^ T-cell depletion, *Candida* overgrowth may be facilitated by direct *Candida*–HIV interactions, including specific binding that could modulate fungal behavior and host responses.

Future research needs to prioritize longitudinal cohort studies, improved fungal sequencing and quantification methods, and integrative multi-omics approaches capable of linking fungal dynamics with bacterial community, host immunity, and clinical outcomes. Advances in human genomics, proteomics, transcriptomics, metabolomics, and artificial intelligence–driven modeling offer promising avenues to overcome current limitations. From a preventive standpoint, insights into bacterial–fungal interactions open doors to new therapeutic strategies, including targeted antifungal approaches, modulation of fungal communities through probiotics or prebiotics, and integrated microbiota-based interventions tailored to restore ecological balance. As research progresses, incorporating fungal-focused perspectives alongside traditional bacterial-centered models may ultimately contribute to more precise, personalized, and effective oral and systemic health care for children.
